# Beyond the snapshot: Landscape genetic analysis of time series data reveal responses of American black bears to landscape change

**DOI:** 10.1111/eva.12617

**Published:** 2018-03-25

**Authors:** Hope M. Draheim, Jennifer A. Moore, Marie‐Josée Fortin, Kim T. Scribner

**Affiliations:** ^1^ Department of Zoology Michigan State University East Lansing MI USA; ^2^ Biology Department Grand Valley State University Allendale MI USA; ^3^ Department of Ecology & Evolutionary Biology University of Toronto Toronto ON Canada; ^4^ Department of Fisheries and Wildlife Michigan State University East Lansing MI USA; ^5^ Department of Integrative Biology Michigan State University East Lansing MI USA

**Keywords:** black bear, landscape change, landscape genetics, time series

## Abstract

Landscape genetic studies typically focus on the evolutionary processes that give rise to spatial patterns that are quantified at a single point in time. Although landscape change is widely recognized as a strong driver of microevolutionary processes, few landscape genetic studies have directly evaluated the change in spatial genetic structure (SGS) over time with concurrent changes in landscape pattern. We introduce a novel approach to analyze landscape genetic data through time. We demonstrate this approach using genotyped samples (*n *= 569) from a large black bear (*Ursus americanus*) population in Michigan (USA) that were harvested during 3 years (2002, 2006, and 2010). We identified areas that were consistently occupied over this 9‐year period and quantified temporal variation in SGS. Then, we evaluated alternative hypotheses about effects of changes in landscape features (e.g., deforestation or crop conversion) on fine‐scale SGS among years using spatial autoregressive modeling and model selection. Relative measures of landscape change such as magnitude of landscape change (i.e., number of patches changing from suitable to unsuitable states or vice versa), and during later periods, measures of fragmentation (i.e., patch aggregation and cohesion) were associated with change in SGS. Our results stress the importance of conducting time series studies for the conservation and management of wildlife inhabiting rapidly changing landscapes.

## 1 INTRODUCTION

Understanding how landscape alteration will influence species distributions and connectivity is the cornerstone to development of successful conservation, restoration, and management strategies (Bolliger, Le Lay, & Holderegger, 2010; Fahrig, 1997). Fragmentation and transformation of natural habitats may change dispersal and colonization potential (Kool, Moilanen, & Treml, 2012). If gene flow is disrupted, increased genetic differentiation (GD) and reduced genetic variation may impact patterns of spatial genetic structure (hereafter SGS) over time (Epperson, 2003). As the field of landscape genetics progresses, it is becoming increasingly clear incorporating landscape features, by assigning resistance values to habitat characteristics, into predictive models (i.e., landscape resistance models) to describe GD is vital to understand which factors impede or facilitate functional connectivity (Spear, Balkenhol, Fortin, McRae, & Scribner, 2010; Storfer, Murphy, Spear, Holderegger, & Waits, 2010). However, further inference about the effects of specific biological and ecological processes that shape SGS is often limited because sampling is typically conducted at a single point in time (Goetze, Andrews, Peijnenburg, Portner, & Norton, 2015). These “snapshots” of data only measure populations in their current state (Anderson et al., 2010; Martensen, Saura, & Fortin, 2017; Schwartz, Luikart, & Waples, 2007). Therefore, inferences can be problematic when studying long‐lived and iteroparous species such as black bears (*Ursus americanus*) that are sensitive to landscape features (i.e., land cover) that are expected to undergo future modification (Cushman, McKelvey, Hayden, & Schwartz, 2006; Cushman, Wasserman, Landguth, & Shirk, 2013). Thus, incorporating a time series approach becomes necessary to make inferences about how a species responds to landscape or environmental change (Sun & Hedgecock, 2017).

Time series data are increasingly being applied to understand biological processes, most notably in the field of ecology and population genetics (Lindenmayer et al., 2012; Schwartz et al., 2007). A number of empirical studies have used temporal genetic data to contrast historical and contemporary genetic diversity (Wandeler, Hoeck, & Keller, 2007). Similarly, there has been increasing interest to include genetic monitoring as an important component of management programs (De Barba et al., 2010; Rudnick, Katzner, Bragin, Rhodes, & Dewoody, 2005; Steele et al., 2013). Assessments of changes in genetic diversity provide a means to evaluate trends in connectivity, to infer demographic histories of populations, and to gauge loss of genetic diversity (Schwartz et al., 2007). However, implementing such studies is challenging because sampling the same population(s) at regular intervals is difficult despite the potential value for conservation and management.

There is increased interest in examining how landscape configuration assessed at different times has influenced contemporary SGS (Landguth et al., 2010; Pavlacky, Goldizen, Prentis, Nicholls, & Lowe, 2009; Zellmer & Knowles, 2009). Yet, few if any, landscape genetic studies have applied a time series approach using multiple genetic and landscape data sets collected at the same time points. Such studies are limited in part by the rarity of multiple samples of the same population from different time points, but are further impeded by the necessity of obtaining complementary time series landscape data. Despite these challenges, time series landscape genetic studies are valuable as natural ecosystems are spatially heterogeneous and landscape composition/configurations can change over time, sometimes drastically so, due to natural and anthropogenic factors (Bolliger et al., 2010; Spear et al., 2010). Furthermore, there may be a discord between the time genetic processes occur (over generations) and the landscape change that reflect contemporary landscape effects (Anderson et al., 2010). Explicitly adding a temporal component in landscape genetics analyses may provide valuable additional resolution on directional trends in gene flow (Martensen et al., 2017; Wagner & Fortin, 2013). By quantifying concurrent changes in SGS and landscape structure over time, we only improve the long‐term predictive power of the effects of ongoing or future landscape change on genetic connectivity (Zellmer & Knowles, 2009).

In this study, we propose a novel approach to quantify changes in SGS through time and relate those changes to underlying landscape change for a population of American black bears in the Northern Lower Peninsula (NLP) of Michigan, USA. The NLP black bear population is bounded to the south by expansive areas of unsuitable human‐dominated urban and agricultural landscapes. The population therefore experiences little to no emigration and immigration. The NLP black bears provide a unique opportunity to apply time series analyses in a landscape context. Indeed, we capitalized on harvest samples acquired over a large extent and long‐term data spanning a 9‐year period. The NLP bear population is subjected to intensive annual harvest (13%–29% of the population is harvested annually, Michigan Department of Natural Resources) indicating the potential for rapid population turnover, and therefore rapid genetic change.

Black bears are affected by forest loss and Michigan's NLP is a heterogeneous landscape that has experienced past and current landscape change (primarily deforestation and agricultural conversion). Hence, we used remotely‐sensed land cover data (Coastal Change Analysis Program, CCAP; https://www.coast.noaa.gov/digitalcoast) that are publicly available every 5 years (2001, 2006, and 2011) for the whole of the NLP during the period over which our genetic samples were collected.

Using a times series approach in a landscape genetic framework, we (i) develop a set of landscape resistance models that incorporate a suite of resistance surfaces representing alternative hypotheses concerning the associations between interindividual genetic distance and landscape resistance distance; (ii) identify and compare the best performing landscape resistance models among years; (iii) evaluate whether local SGS patterns changed over time; and (iv) quantify landscape change and use spatial autoregressive model selection to test for associations between changes in SGS and landscape change.

## 2 METHODS AND MATERIALS

### 2.1 Study area

**Figure 1:**
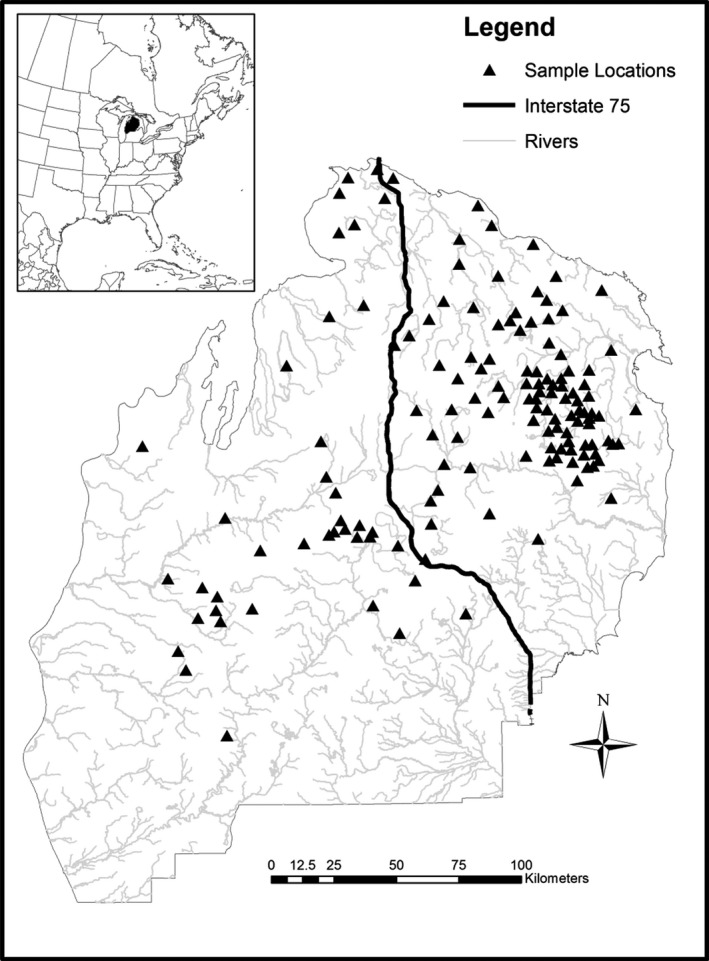
Study area in the Northern Lower Peninsula of Michigan (NLP) showing areas (*n* = 141) of consistent sampling for black bear harvested during 2002, 2006, and 2010 (*n* = 569), interstate‐75 (I‐75) and major rivers

**Figure 2:**
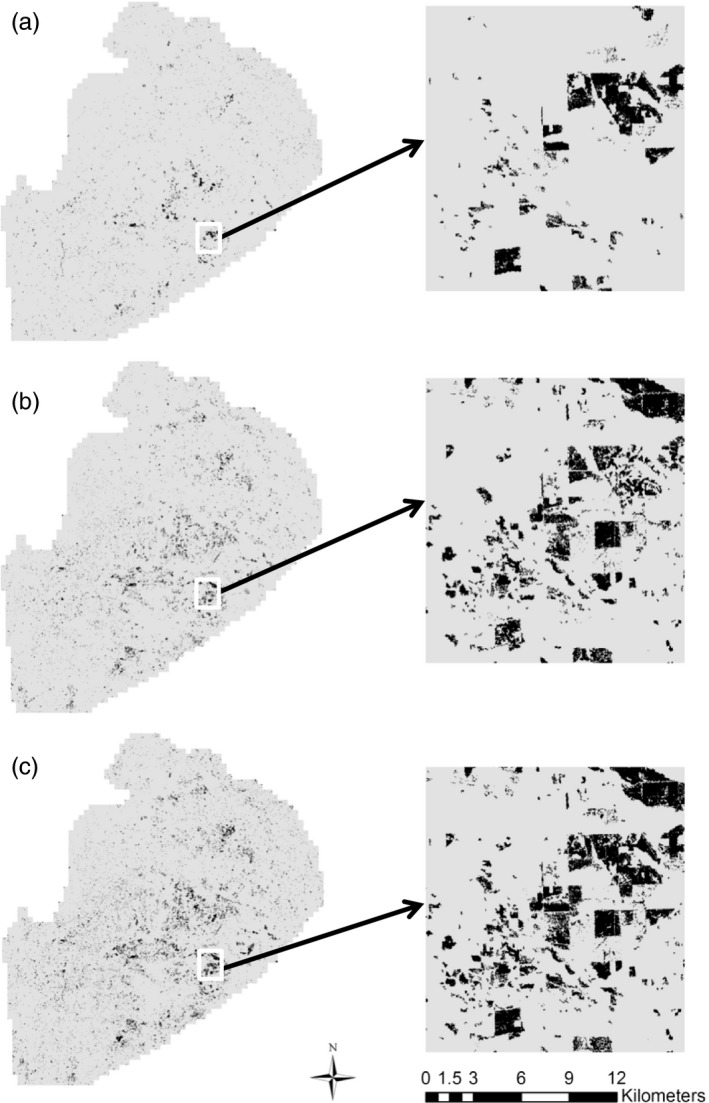
Extent and configuration of forested land lost (gray) over the sampling period from (a) 2001–2006, (b) 2006–2010, and (c) 2002–2010

Our study area covers the northern two‐thirds of the lower peninsula of Michigan (47,739 km^2^) (Figure 1). The NLP is a fragmented mosaic of different land cover and land‐use types including development, agriculture, upland nonforested openings, northern hardwood and mixed hardwood, oak, aspen, pine, forested wetland, and nonforested wetland. The NLP experiences ongoing landscape alteration primarily due to forestry practices and crop conversion resulting in many small fragmented patches of land cover change (Figure 2). Bear sampling occurred during fall harvest (September–October) of 2002 (*n *= 263), 2006 (*n *= 385), and 2010 (*n *= 336). Annually, MDNR requires all harvested bears to be registered at hunter check stations. During registration, a premolar tooth is extracted for aging and DNA extraction (Coy & Garshelis, 1992). Hunters report the bear's harvest location and sex. Locations of bear harvest samples were recorded as township, range, and section, which were then georeferenced to the section centroid and converted into UTM coordinates.

### 2.2 Defining temporal sampling locations

To remove potential sampling error that can arise from differences in dispersion and extent of sampling in opportunistically collected annual harvest samples, we identified areas that were consistently occupied in both space and time. First, for each year (2002, 2006, and 2010), we created Voronoi polygons for all samples within a year. Voronoi polygons are created by partitioning the study site with *n* sampling points into convex polygons, in which a polygon contains only one sampling point and every location in a given polygon is closer to its generating sampling point than any other point (Longley, Goodchild, Maguire, & Rhind, 2010). We then overlaid the three annual Voronoi polygons and used spatial–temporal analysis of moving polygons in ArcGIS 10.1 to define areas where the three polygon layers overlapped and contained at least one bear from each year (Figure S1). We defined these as “areas of consistent sampling” (Figure S2) over the entire (2002–2010) sampling period. Voronoi polygons are used as a way to ensure that throughout the years that subareas (here Voronoi polygons) contain enough samples so that comparisons from 1 year to another is possible. Hence, the Voronoi polygons are not used to estimated/mapped density but rather to delineate subareas such that changes are attributed to more or less the local conditions that the bears were exposed to. Samples within the overlap area of consistent sampling (2002, *n* = 204; 2006, *n* = 199; 2010, *n *= 166) were used in all downstream population and landscape genetic analyses.

### 2.3 Microsatellite genotyping

We extracted DNA from bear teeth using Qiagen DNEasy Tissue Kits (Qiagen Inc., Valencia, CA, USA) following manufacturer protocols. We quantified DNA using a Nanodrop spectrophotometer (Thermo Scientific, Waltham, MA, USA) and diluted samples to a 20 ng/μl working concentration. We used PCR to amplify 12 variable microsatellite loci including: G10X, G10L, G10D, G10B, G10M (PCR annealing temperature TA = 58°C; Paetkau, Calvert, Stirling, & Strobeck, 1995) UarMU59, UarMU50 (TA = 58°C; Taberlet et al., 1997), ABB1, ABB4 (TA = 54°C; Wu, Zhang, & Wei, 2010), UT29, UT35, and UT38 (TA = 54°C; Shih, Huang, Li, Hwang, & Lee, 2009). We amplified DNA according to conditions outlined in Moore, Draheim, Etter, Winterstein, and Scribner (2014). Amplified products were sized on 6.5% denaturing acrylamide gels for electrophoresis and visualized on a LI‐COR 4200 Global IR2 System (LI‐COR Inc., Lincoln, NE, USA). All alleles were scored independently by two experienced laboratory personnel using SAGA genotyping software (LI‐COR Inc.). To assess genotyping error, 10% of samples were randomly selected and genotyped twice to yield a genotyping error rate of <2%.

We used program MICRO‐CHECKER (Van Oosterhout, Hutchinson, Wills, & Shipley, 2004) to test for the presence of null alleles and allelic dropout. We tested for deviations from Hardy–Weinberg equilibrium based on exact tests χ^2^ using program GENEPOP (Version 3.1d; Raymond & Rousset, 1995) and used sequential Bonferroni tests to correct for multiple comparisons (Rice, 1989). We used Bonferroni corrections (Goudet, 1995, 2001) to test for linkage disequilibrium (evaluated by permutation tests, based on 660,000 randomizations) in program FSTAT 2.93. We quantified microsatellite genetic diversity using mean number of alleles (*A*), observed heterozygosity (*H*_O_), and expected heterozygosity (*H*_E_) over all loci, using program GenAlEx (v. 6.0, Peakall & Smouse, 2006). We estimated inter‐individual GD, by calculating the ratio proportion of shared alleles (Dps, GD = (1 − Dps); Bowcock et al., 1994) for each pairwise combination of individuals using GenAlEx v. 6.0 (Peakall & Smouse, 2006). Dps is a commonly used individual‐based genetic distance measure in landscape genetic studies and has been shown to accurately reflect SGS and connectivity at small spatial scales (Murphy, Dezzani, Pilliod, & Storfer, 2010).

### 2.4 Landscape genetic analysis: isolation by distance versus isolation by resistance

We characterized land cover for the three sampling periods using NOAA CCAP Land Cover digital coverage maps, derived from Landsat TM imagery for 2001, 2006, and 2011. To test for isolation by resistance (IBR; to determine how functional connectivity was influenced by landscape features), we generated resistance surfaces for each temporal land cover data set using Spatial Analyst in ArcGIS 10.1. We weighted each land cover class according to positive or negative associations with black bear presence based on habitat suitability indices (HSI) developed independently from telemetry data by Carter, Brown, Etter, and Visser (2010). Landscape resistance surfaces were based on three features (roads, rivers, and land cover) that have previously been reported to influence habitat selection by black bears in the NLP (Carter et al., 2010). Models included resistance surfaces using a land cover classification from CCAP Land Cover datasets (resolution = 150 m, 25 classes). We reclassified datasets into seven land cover classifications according to bear habitat suitability (HSI; cost = 1–100, least to most resistant to bear movement): mixed deciduous forest (MF = 1), forested wetland (FW = 1), evergreen forest (EF = 10), nonforested upland (NFU = 20), agriculture (AG = 50), nonforested wetland (NFW = 100), and developed (DEV = 100). We also included major rivers (10) and roads weighted based on traffic patterns (Interstate‐75 = 100; state roads = 50; all other roads = 10), because they represent putative physical barriers to dispersal. In addition, we created a distance only landscape with a raster surface value of 1 bounded by the same spatial extent as all other resistance surfaces to reflect isolation by distance (IBD). Based on those predictors, we evaluated 11 landscape hypotheses of IBR (Table 1). Using the sum function of raster algebra in Spatial Analyst ArcGIS 10.1, we combined land cover with roads, rivers, and roads + rivers. Resistance distances can be sensitive to the range of relative weights assigned to land cover types. Thus, we performed a sensitivity analysis similar to Draheim, Moore, Etter, Winterstein, and Scribner (2016) using large‐scale and small‐scale resistance weights (i.e., large scale = 1, 10, 20, 50, 100, and small scale = 0.1, 1, 2, 5, 10) and found resistance distances were not sensitive to the scale of land cover resistance weights.

**Table 1:** Mantel (isolation by distance only; IBD) and partial Mantel correlations (*r*) between spatial and genetic pairwise distances among individual black bears in the NLP for 2002, 2006, and 2010. Bold indicates competitive models based on causal modeling

Mantel and partial Mantel test	2002		2006		2010	
	*r*	*p*	*r*	*p*	*r*	*p*
Isolation by distance	.123	.013	.101	.034	.106	.020
Resistance models						
State roads (STR)	−.060	.990	−.027	.672	−.033	.714
Interstate 75 (I75)	−.089	.986	−.013	.566	−.031	.738
All roads	−.043	.806	−.039	.184	−.037	.801
Rivers	−.051	.858	−.009	.509	**.098**	**.037**
Roads + Rivers	−.049	.858	−.032	.752	−.004	.440
Land cover (LC)						
LC cover only	.008	.390	**.214**	**.005**	**.097**	**.031**
LC + Roads	.008	.361	.143	.011	.082	.041
LC + Rivers	.019	.219	.192	.005	**.101**	**.027**
LC + Roads + Rivers	.011	.257	.159	.009	.081	.035
LC + STR	.002	.316	.159	.009	**.088**	**.041**
LC + I75	.026	.200	.187	.005	**.104**	**.026**

STR, State Roads; I75, Interstate‐75; LC, Land cover.

We calculated pairwise resistance distances using CIRCUITSCAPE 3.5 (McRae, 2006). CIRCUITSCAPE incorporates circuit theory to quantify the total landscape resistance between individuals via multiple potential paths of least resistance (McRae & Beier, 2007). We used Mantel (Mantel, 1967) and partial Mantel tests (Smouse, Long, & Sokal, 1986) to correlate genetic and geographic/resistance distances. Legendre and Fortin (2010) noted that Mantel and partial Mantel testing is an appropriate framework when hypotheses are explicitly defined in terms of distances, and are appropriate for individual‐based analyses. The significance of the Mantel and partial Mantel correlations was evaluated by permutation tests, based on 10,000 randomizations. The top candidate model corresponded to the largest partial Mantel *r* values. Next, to eliminate the potential of high Type I error and spurious correlations, we compared a set of competitive IBR models (models with correlation values close to the top model) against each other rather than the null model of IBD. Due to the inherent risk of false positives using Mantel tests, we used an additional analytical framework proposed by Cushman et al. (2013) that is based on the relative support of each candidate model to separate the true resistance model from a range of erroneous alternative resistance models. The relative support for each model was based on the difference in the partial Mantel correlation after partialling out the resistance distance of the other model. The model with significant and positive relative support values for all comparisons was considered the best candidate model (Cushman et al., 2006, 2013).

### 2.5 Landscape–genetic change analysis

#### 2.5.1 Defining landscape change

To quantify the land cover change among time periods, we used the math algebra function in Spatial Analyst ArcGIS 10.1 to calculate the net difference in resistance surface weight from above reclassified annual (2001, 2006, and 2011) CCAP resistance surfaces (resolution 150 m) to reflect change in resistance to black bear movement among time periods. A raster cell with no change in resistance cost was assigned a value of 0, and all other raster cells ranged from −99 to 99. The value and sign (positive or negative) of the raster cell depends on the type of land cover change that occurred from time 1 to time 2 (positive = decreased cost or gain of habitat; negative = increased resistance cost or loss of habitat). For example, if a raster cell at time 1 changed from deciduous forest (low resistance cost to bear movement = 1) to a less suitable habitat of nonforest (higher cost to movement = 20) at time 2, the cell was assigned a landscape change value of −19. Conversely, if a raster cell changed from nonforest to a more suitable habitat like deciduous forest, it was assigned a landscape change value of 19. Correspondingly, if a raster cell in the first scenario changed from deciduous forest (low resistance cost of bear movement = 1) to development (highest resistance cost of bear movement = 100) rather than nonforest, it was assigned a landscape change value of −99, representing the strongest loss of habitat and impediment of movement. Thus, we define land cover change as any change (positive or negative) in a resistance value from time 1 to time 2.

We then overlaid a sampling grid of 2.6 km^2^ cells (representing the approximate area of a section and minimum distance among sampling points) over the land cover change layers. We described landscape change in terms of proportion a sampling grid cell (i.e., 2.6 km^2^) of deciduous forest and/or mixed forest loss (DMFL). The landscape metrics calculated using program FRAGSTATS (McGarigal, Cushman, Neel, & Ene, 2002); definitions of variables and data sources are listed in Table S1) assessed the relative magnitude of change (i.e., NP, PLAND, FL, and DMFL) and change in landscape configuration change (i.e., AI, COH, NNDIST). However, genetic structure can have a substantial time lag in its response to changes in gene flow resulting from a barrier. Furthermore, we also calculated the distance to landscape features that may act as a putative barrier to functional connectivity: (i) distance to major human population center (i.e., city or town; HPDIST); (ii) distance to major road (RDDIST); (iii) distance to major river (RVDIST).

In addition, using ArcGIS 10.1 we calculated the cumulative degree of landscape change (DEG) within each 2.6 km^2^ sampling cell and between neighboring sampling cells (NDEG) that describes the level of landscape change (e.g., forest to development = −99 vs. forest to nonforest land = −19) to test for relative sensitivity of genetic change based on landscape change type within sampling grid cell and of those neighboring a sampling grid cell, respectively. Also, because deforestation is the predominant type of landscape change, we also described landscape change in terms of proportion of area within a sampling grid cell with forest loss (FL).

#### 2.5.2 Defining genetic change

**Figure 3:**
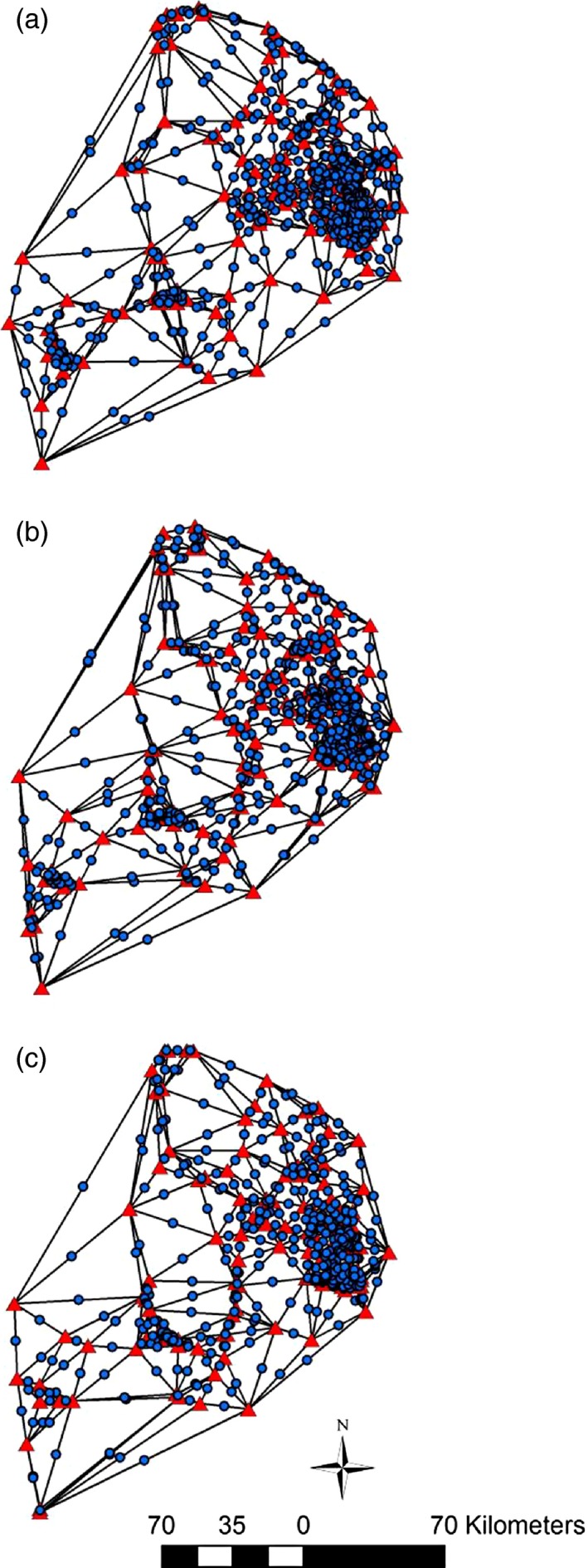
Delaunay triangulation network between individual locations for (a) 2002, (b) 2006, and (c) 2010 used to create genetic surfaces. Red triangles represent sample locations. Blue circles represent midpoints between sampling locations in which genetic differentiation was interpolated

Individual‐based landscape genetic analyses are often limited to link‐based methods. These type of analyses relate pairwise genetic distance between individuals to their landscape distance in which hypotheses are explicitly defined in terms of distances. However, to move beyond link‐based methods we must transform individual‐based pairwise genetic distances into a single Y vector. Landscape genetic neighborhood‐based approaches can use information from pairwise links (genetic distances) to create a node‐based data structure to relate GD patterns to local landscape predictors (James, Coltman, Murray, Hamelin, & Sperling, 2011; Wagner & Fortin, 2013). Here we introduce a novel analytical framework to test whether SGS changed over time. We generated our response variable by producing spatial genetic data layers using the Genetic Landscapes Toolbox (Vandergast, Perry, Lugo, & Hathaway, 2011) in ArcGIS 10.1. This tool constructs a Delaunay triangulation network (Figure 3) between individual locations. At the geographic coordinates of the midpoint of the connecting edges of the network, the program assigns user inputted genetic distance value (i.e., Dps). Then, using Inverse Distance Weighting (power = 2, variable search radius with 12 points), we created an interpolated genetic landscape surface in which each raster cell is weighted by GD among sample locations (Vandergast et al., 2013). To avoid extrapolating beyond the original collection locations, genetic surfaces were clipped to the spatial extent of collection locations. Visual inspection of the three annual genetic surfaces indicated SGS patterns changed between sampling periods. To identify areas where GD may have strengthened or weakened, we calculated the difference in GD estimates among two time periods (i.e., GD at time 1–GD at time 2) using the math algebra function in Spatial Analyst ArcGIS 10.1. We generated two 4‐year and one 8‐year genetic change surface(s) by subtracting the later GD estimate from the earlier GD estimate (4 years = GD_2002_–GD_2006_, GD_2006_–GD_2010_; 8 years = GD_2002_–GD_2010_). Positive values indicate local SGS weakened over time, whereas negative values indicate SGS strengthened over time. We then overlaid the same sampling grid of 2.6 km^2^ cells used to quantify the 12 landscape metrics and quantified our response variable “cumulative genetic change” (GC) by averaging the cumulative difference of GD among years that fell within a sampling grid cell.

#### 2.5.3 Spatial modeling analyses

We compared multiple spatial autoregressive lag models to determine whether landscape change was associated with genetic change. These models are linear regressions that account for spatial nonindependence in the response variable (genetic change) layer with an additional spatial lag term as an explanatory variable and parameter estimation assessed by Maximum Likelihood Estimation (Ward & Gleditsch, 2008). Models without significant regression coefficients (*p* < .05) were discarded. Because of the inherent relationships between landscape change magnitude metrics NP, PLAND, DEG, FL, and DMFL (Table 2), we performed five univariate analyses with each of these explanatory variables and compared them using Akaike's information criterion (AIC) (Burnham & Anderson, 2002; Johnson & Omland, 2004), where Akaike weights represent the probability that a model is the best of the set (Ward & Gleditsch, 2008). The best landscape change magnitude metric of the single regression models was then used in multiple regression analyses. All tests were performed in the *spdep* package in R v. 3.0.1 (R Core Team, 2013).

**Table 2:** Most supported univariate spatial regression models to predict black bear genetic change using landscape variables that characterize the relative magnitude of landscape change in the Northern Lower Peninsula, Michigan, USA. Abbreviations are as described in Table S1

Model (GC ∼)	Intercept	Coeff.	*Z*‐value	ρ	AIC	ΔAIC	wAIC
2002–2006							
**β**_**NP**_	**0.353**	−**.011**	−**2.634**	**.955**	**17,442**	**0**	**0.49**
β_PLAND_	0.212	−.045	−1.125	.955	17,448	6	0.03
β_DEG_	0.194	−.112	−0.734	.955	17,448	6	0.02
*Β*_DMFL_	0.255	−.398	−2.595	.955	17,443	1	0.43
β_FL_	0.213	−.052	−1.266	.955	17,447	5	0.03
2006–2010							
**β**_**NP**_	−**0.613**	**.010**	**2.806**	**.872**	**18**,**667**	**0**	**0.65**
β_PLAND_	−0.470	.092	2.132	.872	18,670	3	0.12
β_DEG_	−0.229	.188	0.792	.871	18,674	7	0.02
*Β*_DMFL_	−0.454	.200	2.249	.871	18,670	3	0.15
β_FL_	−0.412	.091	1.789	.872	18,672	5	0.06
2002–2010							
**β**_**NP**_	**0.007**	**.011**	**0.539**	**.959**	**16**,**535**	**0**	**0.30**
β_PLAND_	0.128	−.025	−1.185	.959	16,536	1	0.23
β_DEG_	0.111	−.098	−0.883	.959	16,537	2	0.17
*Β*_DMFL_	0.103	−.039	−0.776	.959	16,537	2	0.16
β_FL_	0.131	−.032	−1.354	.959	16,537	2	0.14

Coefficients (Coeff) and their corresponding *Z*‐values refer to genetic change layer coefficients, whereas ρ is the spatial lag coefficient. All values of ρ were significant (*p* < .01). AIC, ΔAIC, and weighted (w)AIC values are reported. The best model is in boldface type.

## 3 RESULTS

### 3.1 Population genetic structure

After accounting for spatial differences in sampling among years, we retained 569 individuals within areas continuously occupied for subsequent landscape genetic analyses (2002, *n* = 204; 2006, *n* = 199; 2010, *n* = 166). We did not find evidence for null alleles or allelic dropout. We detected significant departures from HWE for Uar50 in 2002; G10X, G10D, G10B, Uar50, and UT29 in 2006; and G10M in 2010. However, no loci were found to deviate significantly from Hardy–Weinberg or linkage disequilibrium across all time periods (Table S2), so all 12 loci were retained for further analyses. For all loci, expected heterozygosity ranged from 0.62 to 0.94, number of alleles per locus ranged from 6 to 28, and genetic diversity was similar among years (Table S2). Inter‐individual genetic distances were correlated with Euclidean geographic distances for all sampling years (2002, *r* = .123, *p* = .013, 2006, *r* = .101, *p* = .034; 2010, *r* = .106, *p* = .020; Table 1; Figure S3).

### 3.2 Isolation by resistance testing

Landscape genetic analyses revealed discrepancies in landscape resistance model performance among years. Statistically significant landscape resistance models (*p *< .05) were only identified in 2006 and 2010 after partialling out the effects of geographic distance (Table 1). The best‐supported models differed among years. For 2006, the best‐supported model included land cover only (LC) based on the highest partial Mantel correlation (*r* = .214, *p *= .005; Table 1) and relative support. In contrast, the best‐supported resistance model for 2010 included land cover and interstate‐75 (I‐75) as a barrier (partial Mantel, *r* = .104, *p* = .026; Table 1); however, causal modeling revealed this model was not significant (*p* > .05) after partialling out three competing models. Therefore, we are unable to conclude which single model is the best‐supported model for 2010. Equally well supported were three alternative models: (i) LC only (*r* = .097, *p* = .031) (ii); LC + rivers (*r* = .101, *p* = .027); and (iii) LC + state roads (STR) (*r* = .088, *p* = .041; Table 1). All competitive models included land cover, confirming that land cover is important for black bear connectivity in the NLP. In addition, landscape genetic analyses were not sensitive to magnitude of land cover cost values (data not shown).

### 3.3 Genetic change and landscape change

**Figure 4:**
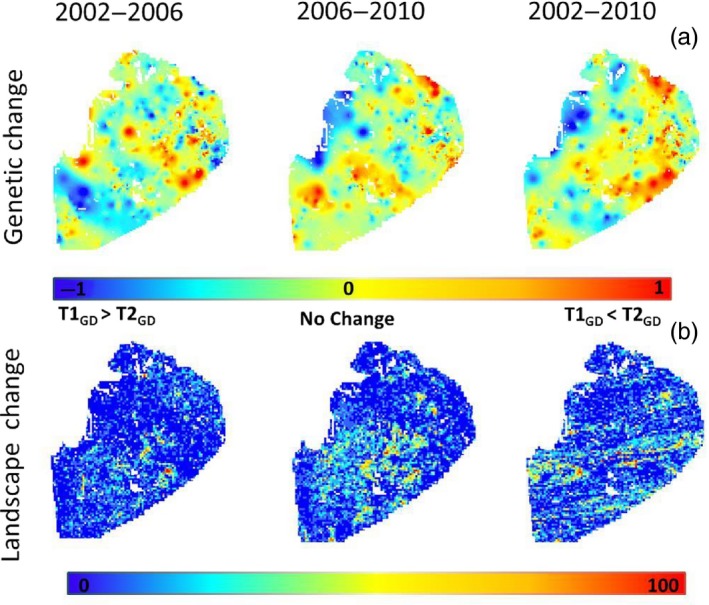
Distribution of (a) the cumulative difference in genetic differentiation (from spatial interpolated genetic surfaces) within a grid cell for all temporal comparisons (2002–2006, 2006–2010, 2002–2010) and (b) number of landscape change patches within a grid cell (2.6 km^2^). For landscape change maps, red indicates a large number of patches of change and blue indicates a low number of patches of change. For genetic change maps, red indicates an increase in genetic differentiation (GD) from time one (T1) to time two (T2) and blue indicates a decrease in genetic differentiation from T1 to T2

The cumulative genetic change surfaces show areas of increased and decreased GD over the study area (Figure 4) indicating local SGS has changed over time. “Landscape change magnitude” univariate modeling predicting genetic change revealed the number of landscape change patches (NP) within a sampling unit was the most supported model for all temporal comparisons (2002–2006, wAIC = 0.49; 2006–2010, wAIC = 0.65; 2002–2010, wAIC = 0.30; Table 2; Figure 4). Therefore, NP was used in multiple regression analyses. Multivariate models with significant (*p* < .05) coefficients are presented in Table 3. All temporal comparisons indicated that genetic change was significantly influenced by habitat alteration calculated as the number of landscape change patches (NP; Table 3; Figure 4). However, for all comparisons that included 2010, genetic change was better predicted by inclusion of landscape change heterogeneity (Table 3). The best model for 2002–2010 included NP and extent of heterogeneity calculated as aggregation (AI; wAIC = 1.0). Similarly, the most probable model in the 2006–2010 comparison included NP, AI, and an additional heterogeneity variable cohesion (COH; wAIC = 0.95 Table 3).

**Table 3:** Most supported multiple spatial autoregression models to predict black bear genetic change using landscape metrics that characterize the relative magnitude or configuration of landscape change in the Northern Lower Peninsula, Michigan, USA. Abbreviations are as described in Table S1

Model (GC ∼)	ρ	AIC	ΔAIC	wAIC
2002–2006				
**β**_**NP**_	**.955**	**16**,**245**		
2002–2010				
**β**_**NP**_ + **β**_**AI**_	**.934**	**16**,**533**	**0**	**1**
β_NP_	.959	16,537	4	0
2006–2010				
**β**_**NP**_ +* * **β**_**AI**_ + **β**_**COH**_	**.900**	**16**,**683**	**0**	**0.95**
β_NP_ + β_AI_	.951	16,689	6	0.05
β_NP_	.872	16,886	203	0
β_AI_ + β_COH_	.945	16,899	216	0
β_AI_	.945	17,001	318	0

ρ is the spatial lag coefficient. All values of ρ were significant (*p* < .01). AIC, ΔAIC, and weighted (w)AIC values are reported. The best model is in boldface type.

## 4 DISCUSSION

The field of landscape genetics can advance beyond characterizing static landscape pattern–genetic process relationships (Balkenhol et al., 2009). Incorporating landscape change and quantifying the effects of change on SGS over time is vital to improve understanding of anthropogenic impacts on functional connectivity (Bolliger et al., 2010; Segelbacher et al., 2010; Storfer et al., 2010). Our study illustrates the advantages of joint use of time series genetic and landscape data when using established landscape genetics methods. Using a long‐term genetic data set and land cover imagery covering an analogous time period, we found disparities in landscape resistance models during different “snapshots” of time (Table 1). Our results show that if landscape does not appear to be an important component shaping functional connectivity at one time point, it does not mean that attributes will not become important over relatively short time scales in changing landscapes. Our findings have broad implications for conservation and management, and speak to the importance of collecting long‐term data.

Black bears in Michigan's NLP exhibit an IBD pattern of gene flow, commonly documented in black bears (Costello, Creel, Kalinowski, Vu, & Quigley, 2008; Moore et al., 2014; Rogers, 1977, 1987; Schwartz & Franzmann, 1992). Indeed, strong IBD is consistent with studies on bears and other wide‐ranging carnivores (Brown, Hull, Updike, Fain, & Ernest, 2009; Paetkau, Waits, Clarkson, Craighead, & Strobeck, 1997; Rueness, Jorde, et al., 2003; Rueness, Stenseth, et al., 2003). However, we found isolation by landscape resistance was more strongly supported than IBD in 2006 and 2010. Land cover was the landscape factor most strongly correlated with inter‐individual genetic distance in black bears in the NLP after partialling out geographic distance (Table 1). In contrast, roads and rivers alone did not appear to influence genetic distance. However, the model that only included rivers was significantly correlated with GD in 2010. Our results are consistent with a previous study that performed similar landscape genetic analyses on NLP black bears sampled during 2006 but used a much larger sample size (*n* = 365) and spatial distribution (Draheim, 2015). The influence of land cover is not surprising as land cover is fundamentally tied to resources such as food availability and forest cover for security and resting (Amstrup & Beecham, 1976; Davis, Weir, Hamilton, & Deal, 2006; Noyce & Garshelis, 2011; Rogers, 1977). Carter et al. (2010) found a negative association between bear location and small and medium roads in the NLP black bear population based on radiotelemetry locations from 1991 to 2000 for 20 males and 35 females. However, our results suggest roads do not unduly influence functional connectivity. Indeed, while previous landscape genetic studies have found roads as a limiting factor to gene flow in black bears, the relative influence of roads varies among populations (Cushman et al., 2006; Short Bull et al., 2011), and in some cases, roads may serve to facilitate rather than impede bear movement (Balkenhol & Waits, 2009).

The cumulative effects of landscape alteration over increasing lengths of time explain the differences in relative effects of landscape features on SGS in NLP black bears. We found significant effects of landscape change on changes in levels of GD (Tables 2 and 3). However, the amount of variation explained in models that included variables associated with habitat heterogeneity were the best predictors of genetic change for temporal comparisons that included 2010 (Table 3). This discrepancy among years reflects an increasing amount of habitat fragmentation due to deforestation over the 9‐year sampling period. Currently, the NLP is a patchy landscape with areas of deciduous, mixed, and coniferous forest fragmented by areas of intensive agriculture or human development. If current patterns of human land‐use continue, the remaining forested areas could undergo further modification in the near future (Public Service Consultants 2001). Based on our landscape change raster layers, approximately 1.3% of black bear habitat is lost every 5 years (2002–2006 = 0.9%, 2006–2010 = 1.6%; Figure 2). On the whole, this habitat loss may not seem extensive; however, the effects of habitat loss can be amplified by concurrent fragmentation (Fahrig, 1997) resulting in an increase in dispersed, complex habitat patches.

Lack of an association between land cover and SGS in bears during 2002 (Table 1) could indicate the degree of land cover heterogeneity present in 2002 was insufficient to cause a response. However, stochastic processes such as sampling variance among years or genetic drift may be contributing factors as well. Our result is consistent with a study in the Rocky Mountains of the United States that evaluated the influence of forest cover on black bear gene flow based on 12 landscapes that varied in degree of fragmentation. Short Bull et al. (2011) found higher correlations between land cover and black bear gene flow in landscapes where forest cover was highly fragmented compared to landscapes of contiguous forest. Yet, the absence of a landscape effect on 2002 SGS may reflect a time lag between when landscape change occurs and when SGS response to landscape change becomes evident (Anderson et al., 2010; Epps & Keyghobadi, 2015). However, when dispersal rates and distances are large, as exhibited in the NLP black bear population (Draheim, 2015; Draheim et al., 2016; Moore et al., 2014), shorter or no time lags are expected (Epps & Keyghobadi, 2015). Also, legacy effects of historical landscape processes may be reduced using genetic markers with higher mutations rates (i.e., microsatellites) that reach mutation–drift equilibrium quickly and genetic measures that respond rapidly to changes in connectivity (e.g., Dps) (Anderson et al., 2010). A number of simulation studies have found landscape effects on SGS could be detected with relatively short time spans (Cushman & Landguth, 2010; Landguth et al., 2010; Murphy, Evans, Cushman, & Storfer, 2008).

Field sampling of wildlife populations can never be conducted exhaustively nor can spatial coverage be completely replicated during every time period. Thus, stochastic sampling processes, especially in populations where density varies across the landscape, can never really be removed from empirical data sets. Harvest data are opportunistically collected, meaning locations will inevitably vary among temporal periods and may be predisposed to erroneous inferences across years (Schwartz & McKelvey, 2009). In our study system, bear harvest samples do show consistent regional density and distribution patterns among years (Draheim, Lopez, Winterstein, Etter, & Scribner, 2015). We additionally controlled for spatial sampling heterogeneity among years by only including areas where bears were consistently harvested over time. We acknowledge that reducing our sample size in this way may reduce statistical power and add sampling noise, but we weighed the benefit of increased sample size against increased spatial sampling bias. Regardless, our samples sizes after accounting for spatial heterogeneity are respectable (*n* = 166–204). Also, Graves et al. (2012) showed that samples located at different locations within a home range did not affect the genetic–landscape relationship, we used a comparable approach and assumption here in this study.

There can be a disconnect between what is statistical vs. biologically significant. Thus, we tried to account for this in our study. First, we based landscape resistance weights on field data from radiotelemetry. Second, we performed a sensitivity analyses on our resistance weights to ensure landscape genetic results were not unduly influenced by the scale of resistance values. Lastly, due to the inherent risk of false positives using Mantel and partial Mantel tests, we implemented an analytical framework proposed by Cushman et al. (2013) that is based on the relative support of each candidate model to separate the true resistance model from a range of erroneous alternative resistance models.

Our finding of genetic change between periods within localized areas over a 9‐year sampling period (Figure 4) was unexpected, due to the longevity and generation time (NLP black bear population generation time = 6.53 years, Waples et al., in press) of black bears. Black bears in the NLP may be experiencing rapid rates of population turnover and subsequent local population fluctuations due to intensive annual harvest (~13%–29% of the population harvested annually between 2002 and 2010, MDNR unpublished data). The juxtaposition of areas of increased and decreased GD over the study area (Figure 4) could reflect these local population fluctuations. The observed patchy distribution of “hot” and “cold” areas of genetic change shown in Figure 4 could be attributed in part to the type of landscape change occurring. Although deforestation is the predominant source of landscape change, there are patches of forest regeneration on the landscape that may facilitate gene flow.

We are unsure as to how genetic drift, which is likely occurring, contributed to our results. If genetic drift was the dominant force contributing to temporal variation in SGS of NLP black bears, we would predict little evidence for IBD/resistance or associations between genetic and landscape change, which is inconsistent with our results. Furthermore, in an graph theory framework, Draheim et al. (2016) found black bears in the NLP exhibit asymmetric gene flow based on areas of high and low net flux indicating source–sink dynamics (Pulliam, 1988). Therefore, if gene flow is mediated by use of corridors among source and sink areas and corridors are disrupted due to landscape change, local SGS is expected to change. Further investigations, for example, using local harvest abundance or other proxies for bear density (e.g., Moore et al., 2014), are needed to address this question.

While our time series approach may be broadly applicable across a range of taxa, the approach needs to be parameterized based on specific life history and landscape characteristics of the species or population of interest. Our findings, while supported in Michigan black bears in the NLP, should be evaluated empirically for other species and locales. For example, we would predict similar genetic/landscape change results based on the degree of concordance between our results and previous landscape genetic studies of black bears in the Rocky Mountains (Short Bull et al., 2011). However, there are a number of possible factors that could confound results in another bear population. For example, the NLP is an isolated population; thus, we could assume changes in SGS were not due to immigration or emigration. Also, it is important that the rate of landscape change be sufficient to influence SGS over relatively short time intervals.

To our knowledge, our study provides the first time series landscape genetic analyses performed across multiple generations of the same population. Here we have shown the importance of time series data to test for consistencies in landscape–genetic relationships over time. Our ability to relate gene flow to landscape features is largely dependent on the temporal scale of sampling. Our finding that genetic change is associated with landscape change highlights the synergistic effects of habitat loss and fragmentation on black bear gene flow. Our data enable managers to target regions or habitat types that are important for maintaining connectivity across anthropogenically altered habitats and assess the impacts of future landscape change.

## DATA ACCESSIBILITY

Data used in this study, including bear microsatellite genotypes and harvest locations, are available for download on Dryad (https://doi.org/10.5061/dryad.c61q0).

## AUTHOR CONTRIBUTIONS

All authors contributed to the theoretical foundation and writing of the manuscript. HD and JM generated data and HD performed analyses.

## CONFLICT OF INTEREST

None declared.
