# Supplementary material for: Beyond the snapshot: Landscape genetic analysis of time series data reveal responses of American black bears to landscape change
Source: Evol Appl. 2018 Mar 25;11(8):1219–30. doi: 10.1111/eva.12617 (PMC6100183; doi:10.1111/eva.12617)

Table S1. A summary of the landscape metrics used to characterize genetic change between 2002, 2006, and 2010 for black bears in the Northern Lower Peninsula of Michigan, USA.

|  |  |  |  |  |
| --- | --- | --- | --- | --- |
| **Variable** | | **Abbreviation** | **Description** | **Program** |
|  |  |  |  |  |
|  |  |  |  |  |
| Magnitude of Landscape Change | |  |  |  |
|  | Quantity of Change | NP | Total number of landscape change patches | FRAGSTATS |
|  | Percent Area of Change | PLAND | Percent of area occupied by landscape change patches | FRAGSTATS |
|  | Degree of Change | DEG | Cumulative difference in landscape change values for all landscape change patches | ArcGIS |
|  | Deforestation | FL | Percent of area occupied by landscape change classified as forest loss. | FRAGSTATS |
|  | Deforestation (deciduous or mixed) | DMFL | Percent of area occupied by landscape change classified as the loss of deciduous or mixed forest only | FRAGSTATS |
| Neighborhood | |  |  |  |
|  | Nearest Neighbor Distance | NNDIST | For a given landscape change path the distance to nearest landscape change patch | FRAGSTATS |
|  | Nearest Neighborhood Change | NDEG | Cumulative difference in landscape change values for all landscape change patches in neighboring sampling cells | ArcGIS |
| Heterogeneity of Landscape Change | |  |  |  |
|  | Aggregation | AI | Extent to which land cover change patches are aggregated | FRAGSTATS |
|  | Cohesion | COH | The physical connectedness of landscape change patches | FRAGSTATS |
| Distance to Barriers | |  |  |  |
|  | Human Population (city) | HPDIST | Distance to nearest human population center | ArcGIS |
|  | Roads | RDDIST | Distance to nearest major road | ArcGIS |
|  | Rivers | RVDIST | Distance to major river | ArcGIS |
|  |  |  |  |  |

Table S2. Black bear genetic diversity at 12 microsatellite loci. Loci that show significant deviations from Hardy-Weinberg equilibrium ([Raymond & Rousset 1995](#_ENREF_45)) are in bold. *Na*, number of alleles per locus; *Ho*, observed heterozygosity; *H_E_*, expected heterozygosity; F_IS,_ inbreeding coefficient.

|  | |  |  |  |
| --- | --- | --- | --- | --- |
|  |  |  |  |  |
| **2002 (n = 204)** | | | | |
| **Locus** | **Na** | **H_O_** | **H_E_** | **F_IS_** |
|  |  |  |  |  |
| G10X | 8 | 0.658 | 0.617 | -0.0417 |
| G10M | 9 | 0.744 | 0.757 | 0.01018 |
| G10D | 9 | 0.776 | 0.752 | -0.0318 |
| G10L | 10 | 0.892 | 0.856 | -0.0377 |
| G10B | 6 | 0.709 | 0.737 | 0.01489 |
| **Uar50** | **8** | **0.756** | **0.783** | **0.04064** |
| Uar59 | 6 | 0.688 | 0.707 | 0.03822 |
| ABB1 | 8 | 0.710 | 0.711 | -0.0008 |
| ABB4 | 7 | 0.789 | 0.795 | -0.0011 |
| UT35 | 6 | 0.760 | 0.771 | -0.0021 |
| UT38 | 21 | 0.951 | 0.908 | -0.0374 |
| UT29 | 12 | 0.859 | 0.844 | -0.0155 |
|  |  |  |  |  |
|  |  |  |  |  |
| **(2006 n = 199)** | | | | |
| **Locus** | **Na** | **H_O_** | **H_E_** | **F_IS_** |
|  |  |  |  |  |
| **G10X** | **15** | **0.668** | **0.702** | 0.048 |
| G10M | 10 | 0.793 | 0.787 | -0.009 |
| **G10D** | **11** | **0.790** | **0.762** | **-0.038** |
| G10L | 10 | 0.818 | 0.860 | 0.049 |
| **G10B** | **8** | **0.715** | **0.727** | **0.016** |
| **Uar50** | **10** | **0.656** | **0.787** | **0.167** |
| Uar59 | 9 | 0.667 | 0.677 | 0.015 |
| ABB1 | 9 | 0.689 | 0.737 | 0.066 |
| ABB4 | 8 | 0.823 | 0.798 | -0.031 |
| UT35 | 7 | 0.773 | 0.784 | 0.014 |
| UT38 | 28 | 0.888 | 0.898 | 0.012 |
| **UT29** | **12** | **0.850** | **0.816** | **-0.041** |
|  |  |  |  |  |
|  |  |  |  |  |
| **2010 (n = 166)** | | | | |
| **Locus** | **Na** | **H_O_** | **H_E_** | **F_IS_** |
|  |  |  |  |  |
| G10X | 9 | 0.776 | 0.721 | -0.061 |
| **G10M** | **10** | **0.759** | **0.765** | **0.018** |
| G10D | 11 | 0.735 | 0.754 | 0.021 |
| G10L | 12 | 0.878 | 0.862 | -0.028 |
| G10B | 6 | 0.680 | 0.716 | 0.073 |
| Uar50 | 9 | 0.747 | 0.816 | 0.067 |
| Uar59 | 7 | 0.721 | 0.691 | -0.008 |
| ABB1 | 10 | 0.738 | 0.742 | 0.008 |
| ABB4 | 10 | 0.822 | 0.790 | -0.021 |
| UT35 | 7 | 0.753 | 0.776 | 0.039 |
| UT38 | 22 | 0.937 | 0.903 | -0.029 |
| UT29 | 11 | 0.911 | 0.941 | 0.075 |
|  |  |  |  |  |

Figure S1. Example of area of consistent sampling determination. A) Voronoi tessellations for 2002 (circles), 2006 (triangles), and 2010 (square) samples, over the same area and B) resulting polygon layer when the Voronoi are overlaid. An area of consistent sampling is defined as an overlapping polygon (gray) that contains at least one sample from each sample year.


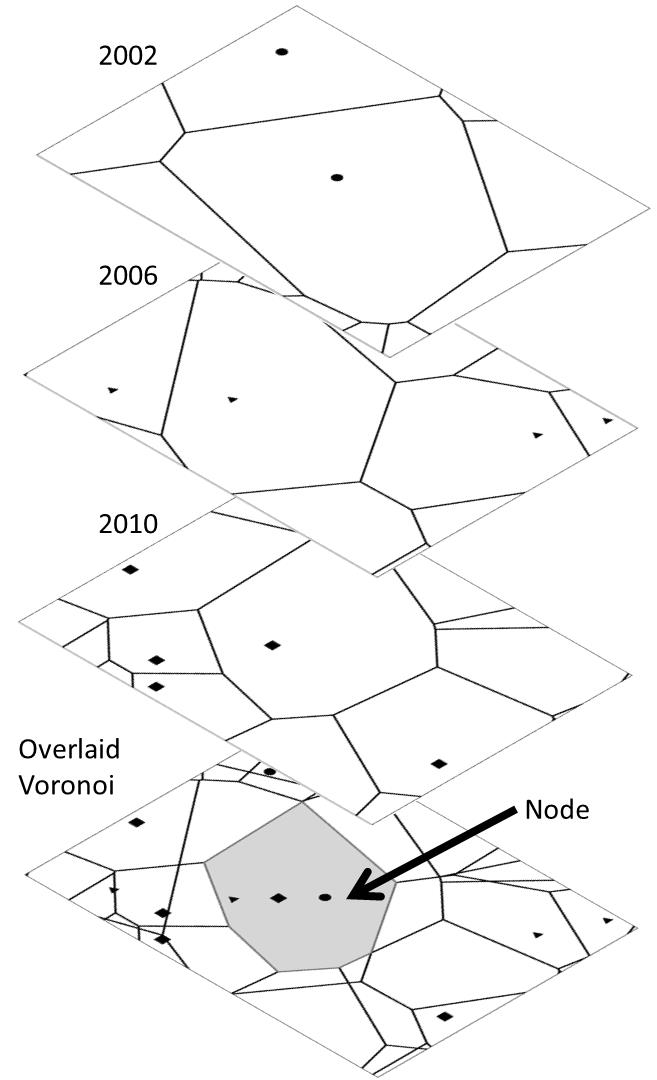


Figure S2. Map of overlapping Voronoi tessellation point pattern polygons in NLP. Polygons contained at least one sample from 2002, 2006, and 2010.


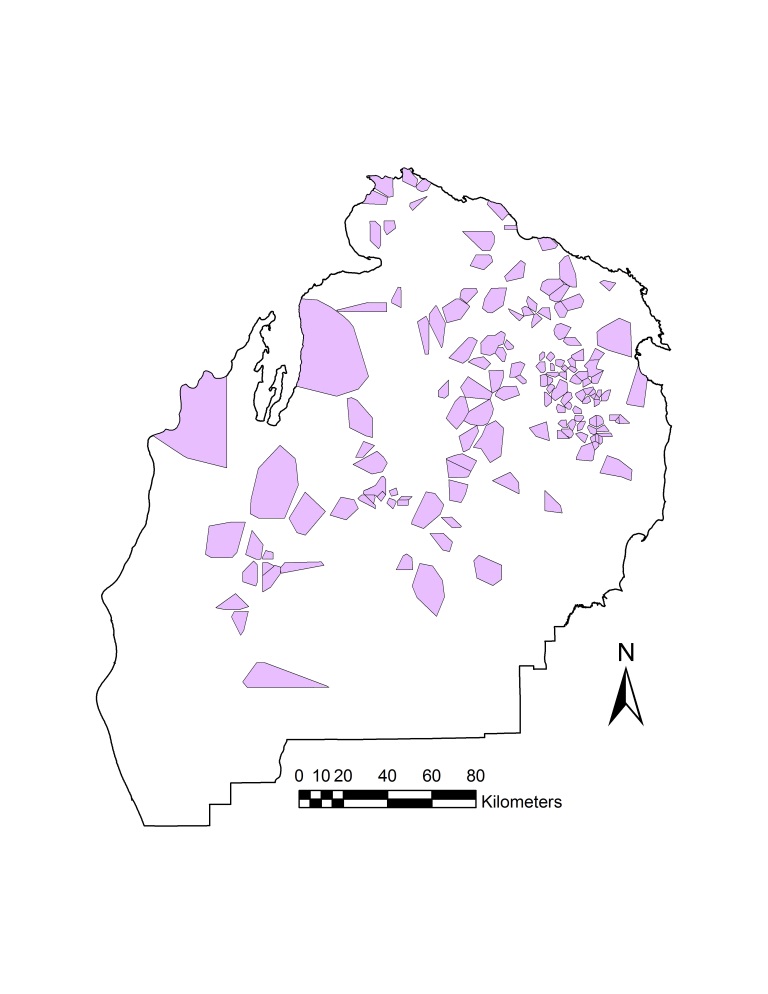


Figure S3. Scatterplot of genetic distance versus geographic distance including least squares regression line (2002, *P* = 0.01; 2006, *P* = 0.03; 2010, *P* = 0.02; Mantel test). Genetic distance was based on pairwise estimates of proportion of shared alleles (Dps) among all individuals. Geographic distances (km) were calculated as the straight-line distance from the center of each sampling location.


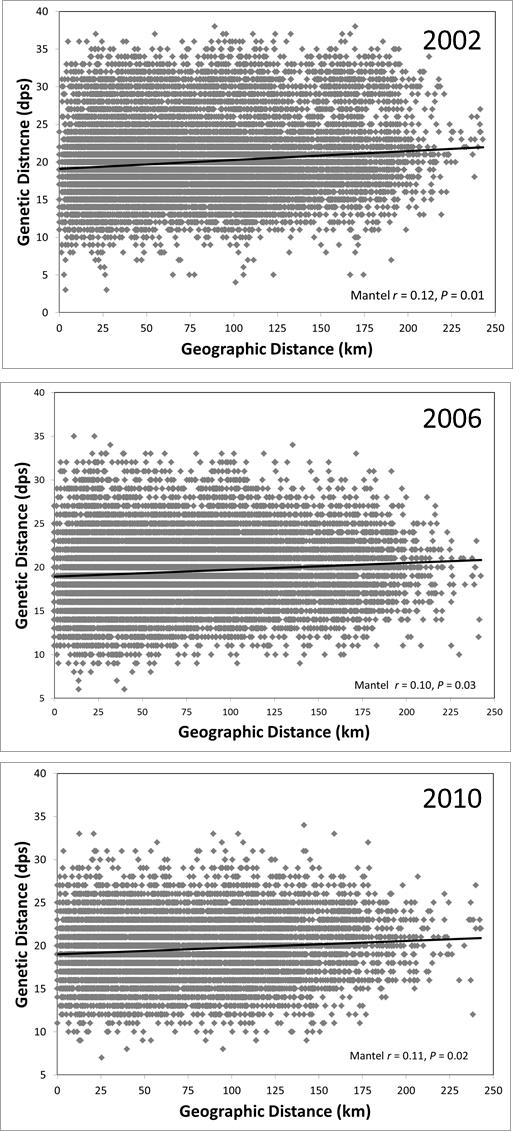

Supplement: Supplementary file 1 [file EVA-11-1219-s001.docx]
